# Renal safety of lithium in HIV-infected patients established on tenofovir disoproxil fumarate containing antiretroviral therapy: analysis from a randomized placebo-controlled trial

**DOI:** 10.1186/s12981-017-0134-2

**Published:** 2017-02-04

**Authors:** Eric H. Decloedt, Maia Lesosky, Gary Maartens, John A. Joska

**Affiliations:** 1 Division of Clinical Pharmacology, Department of Medicine, Faculty of Medicine and Health Sciences, Stellenbosch University, PO Box 241, Cape Town, 8000 South Africa; 2 Division of Neuropsychiatry, Department of Psychiatry and Mental Health, Faculty of Health Sciences, University of Cape Town, Cape Town, South Africa; 3 Division of Epidemiology and Biostatistics, School of Public Health and Family Medicine, University of Cape Town, Cape Town, South Africa; 4 Division of Clinical Pharmacology, Department of Medicine, Faculty of Health Sciences, University of Cape Town, Cape Town, South Africa; 5 Department of Medicine, Faculty of Health Sciences, University of Cape Town, Cape Town, South Africa

**Keywords:** South Africa, Randomised placebo-controlled clinical trial, HIV-associated neurocognitive impairment, HIV, Lithium, Placebo, Antiretroviral therapy, Tenofovir

## Abstract

**Background:**

The prevalence of bipolar disorder in HIV-infected patients is higher than the general population. Lithium is the most effective mood stabiliser, while tenofovir disoproxil fumarate (TDF) is frequently used as part of combination antiretroviral therapy (ART). Both TDF and lithium are associated with renal tubular toxicity, which could be additive, or a pharmacokinetic interaction may occur at renal transporters with a decrease in TDF elimination.

**Objective:**

We report on the change in estimated glomerular filtration rate (eGFR) using the modification of diet in renal disease formula in participants who received ART including TDF and were enrolled in a 24 week randomised trial of lithium versus placebo in patients with HIV-associated neurocognitive impairment.

**Methods:**

We included HIV-infected adults with cognitive impairment established on ART for at least 6 months with a suppressed viral load attending public sector ART clinics in Cape Town, South Africa. We excluded participants with an eGFR <60 mL/min and treated with medications predisposing to lithium toxicity. We reviewed participants weekly for the first month for adverse events followed by 4 weekly visits for renal function assessment, adverse event monitoring and adherence. Lithium dose was titrated to achieve the maintenance target plasma concentration of between 0.6 and 1.0 mmol/L. Sham lithium concentrations were generated for participants receiving placebo.

**Results:**

We included 23 participants allocated to the lithium arm and 30 participants allocated to the placebo arm. Baseline characteristics were not statistically different with a mean age of 37.7 and 40.8 years, a median time on ART of 33 and 40 months and an eGFR of 139.3 and 131.0 mL/min in the lithium and placebo arms respectively. There was no statistical significant difference in the reduction in eGFR or increase in potassium between the two arms during the 24 weeks.

**Conclusions:**

We found that 24-week treatment of HIV-infected patients with lithium and TDF did not result in increased nephrotoxicity.

*Trial registration* The study was registered on the Pan African Clinical Trials Registry (PACTR) with the identifier number PACTR201310000635418. Registered 11 October 2013 before the first participant was enrolled

## Background

The prevalence of bipolar disorder in human immunodeficiency virus (HIV)-infected patients is 4–5 times higher than the general population [1, 2]. The most effective mood stabiliser is lithium while the nucleotide reverse transcriptase inhibitor tenofovir disoproxil fumarate (TDF) is frequently used as part of combination antiretroviral therapy (ART) [3]. Both TDF and lithium are associated with renal tubular toxicity, which could be additive [4, 5]. Furthermore, a TDF-lithium pharmacokinetic interaction may occur at renal transporters. TDF is eliminated via proximal tubular secretion and renal toxicity is thought to be related to accumulation of intracellular tenofovir in the proximal tubular cell [6]. Intracellular TDF inhibits mitochondrial deoxyribonucleic acid (DNA) polymerase gamma with DNA depletion and oxidative respiratory chain dysfunction [7]. Mitochondrial dysfunction impairs tubular reabsorption of ions and molecules causing a Fanconi-like syndrome, or may lead to cell apoptosis and acute tubular necrosis [7]. TDF is a substrate of a number of transporters at the proximal renal tubule. The organic anion transporter-1 (OATP-1) transports TDF intracellularly while the multi-drug resistance protein 4 (MRP-4) mediates active secretion from the tubular cell [8]. In rats lithium impairs OATP-1 function, which may protect against TDF renal toxicity [9]. Twenty to eighty seven percent of lithium treated patients develop a reduction in urinary concentrating ability within weeks after starting treatment [10]. Lithium-induced nephrogenic diabetes insipidus (NDI) is thought to be caused by downregulation of intracellular calcium signalling with inhibition of glycogen synthase kinase-3-beta (GSK-3-ß), resulting in a number of downstream effects including decreased aquaporin-2 expression [11]. Recently it was shown that MRP-1 expression is regulated by GSK-3-β, suggesting that lithium may decrease MRP expression and predispose to TDF renal toxicity [12]. The proximal tubule as a common site for TDF and lithium renal toxicity may further contribute to renal toxicity. Lithium-induced renal toxicity may involve any segment of the nephron or kidney although the distal tubule seems to be involved in NDI [13]. There is currently no published data on the renal safety of concomitant TDF and lithium.

We previously published the results of a 24 week randomised placebo-controlled trial to study lithium as an adjunctive pharmacotherapy in patients with moderate to severe HIV-associated neurocognitive impairment [14]. In this study we report changes in the estimated glomerular filtration rate (eGFR) using the modification of diet in renal disease (MDRD) formula as well as changes in potassium in participants who received ART including TDF who were randomised to lithium or placebo.

## Methods

Our methodology has been previously published, but in brief we included HIV-infected adults (≥18 and ≤70 years), established on ART for at least 6 months with a suppressed viral load (HIV RNA <400 copies/mL) with cognitive impairment attending public sector ART clinics in Cape Town, South Africa [14]. We dosed lithium carbonate 250 mg tablets (Camcolit^®^, manufactured by Norgine) and matching placebo (manufactured by Norgine). We excluded participants who used medications that may predispose to lithium toxicity (diuretics, angiotensin converting enzyme inhibitors or angiotensin receptor blockers and non-steroidal anti-inflammatory medicines), participants with an eGFR of less than 60 mL/min and dehydrated participants with diarrhoea. We reviewed participants weekly for first month followed by 4 weekly visits for adverse events and adherence. After screening and study drug initiation, we repeated renal function (sodium, potassium, urea and creatinine) at weeks 4, 8, 12, 16, 20 and 24. Some participants switched treatment to TDF during the study period, and for this analysis we only included patients who received TDF for the full 24 weeks. Lithium dose was titrated assuming linear pharmacokinetics to achieve the maintenance target plasma concentration of lithium in patients with bipolar mood disorder of between 0.6 and 1.0 mmol/L. Sham lithium concentrations were generated for participants receiving placebo by the study statistician who was unblinded to treatment allocation. After each visit an investigator not directly responsible for participant follow-ups received a log with the participant number, blinded lithium concentration from the study statistician (real or sham), current study drug dose and any adverse events noted by other investigators. Based on the information the investigator recommended lithium and sham dose adjustments for implementation.

## Results

**Fig. 1:**
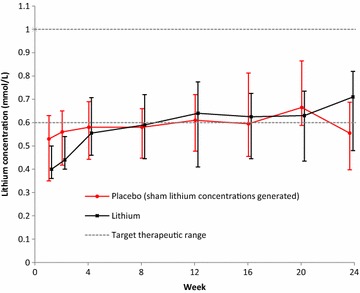
The graph shows the mean change in lithium concentrations. The *bars* indicate median and interquartile range change in lithium concentrations over the 24 weeks in the lithium and placebo arms respectively. Sham lithium concentrations were generated for the placebo arm. The *broken line* indicates the target therapeutic range of 0.6–1.0 mmol/L

**Fig. 2:**
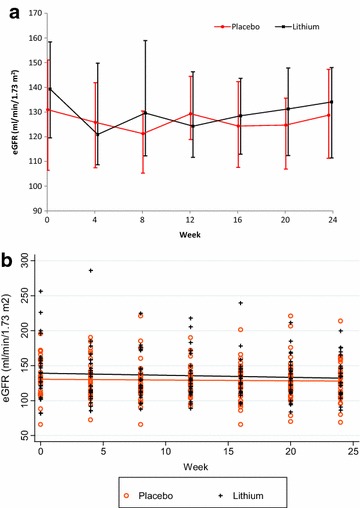
**a** The graph shows the mean estimated glomerular filtration rate (eGFR) as calculated using the modification of diet in renal disease (MDRD). The *bars* indicate median and interquartile range change in eGFR over the 24 weeks in the lithium and placebo arms respectively. **b** Scatter plot of estimated glomerular filtration rate (eGFR) as calculated using the modification of diet in renal disease (MDRD) over the 24 weeks in the lithium and placebo arms respectively. The *solid lines* indicate the linear regression lines for the lithium and placebo arms respectively. Treatment allocation did not have a statistically significant effect on eGFR (p value 0.06)

**Fig. 3:**
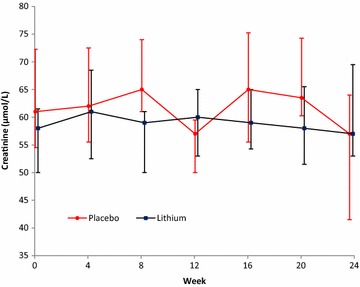
The graph shows the mean change in creatinine (µmol/L). The *bars* indicate median and interquartile range of creatinine over the 24 weeks in the lithium and placebo arms respectively

**Fig. 4:**
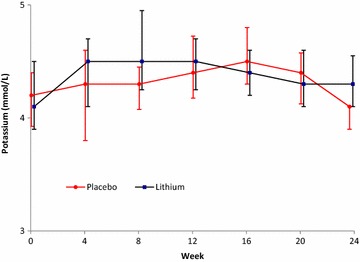
The graph shows the mean change in potassium. The *bars* indicate median and interquartile range change in potassium over the 24 weeks in the lithium and placebo arms respectively

We included 53 participants in this analysis with 23 participants allocated to the lithium arm and 30 participants allocated to the placebo arm. Baseline characteristics between the 2 arms were similar and are described in Table 1. Adherence in both treatment arms were similar and reported previously [14]. The proportion of patients with lithium concentrations in the therapeutic range are presented in Fig. 1. Three participants allocated to the lithium arm developed symptoms of NDI (polyuria) which resolved on dose reduction (p = 0.042 compared to placebo arm). No participant allocated to the placebo arm developed symptoms of NDI. Change in eGFR, creatinine and potassium were similar between the 2 arms (see Figs. 2, 3, 4). There was no statistical significant difference between the two arms in the proportion of participants who had a reduction in eGFR (see Table 2). There was no statistically significant difference in the eGFR slope between the 2 treatment arms (see Fig. 2a) (p value = 0.06) when using linear regression.

**Table 1:** Baseline characteristics

Baseline characteristic	Lithium (n = 23)	Placebo (n = 30)	p value
Gender			
Male	n = 1 (4%)	n = 4 (13%)	0.374^e^
Female	n = 22 (96%)	n = 26 (87%)	
Age	37.7 ± 8.1^a^ years	40.8 ± 8.54^a^ years	0.186^c^
Weight	68.5 ± 16.2^a^ kg	71.2 ± 12.7^a^ kg	0.493^c^
Months on ART	33 (12–56)^b^ months	40 (26–68)^b^ months	0.262^d^
Renal function			
Creatinine	58 (49–62)^b^ µmol/L	58.5 (50–68)^b^ µmol/L	0.404^d^
eGFR MDRD	139.3 (118.1–159.7)^b^ mL/min	131.0 (110.9–156.9)^b^ mL/min	0.572^d^

*eGFR* estimated glomerular filtration rate, *MDRD* modification of diet in renal disease formula

^a^Mean and standard deviation

^b^Median and interquartile range

^c^t-test (2 samples)

^d^Wilcoxon sum rank

^e^Fisher’s exact test

**Table 2:** Estimated glomerular filtration rate change

eGFR MDRD change^d^	Treatment arm	Week 4 % (n)	Week 8 % (n)	Week 12 % (n)	Week 16 % (n)	Week 20 % (n)	Week 24 % (n)
Increased	Lithium	30.4% (7/23)	39.1% (9/23)	39.1% (9/23)	34.8% (8/23)	39.1% (9/23)	56.5% (13/23)
	Placebo	40% (12/30)	50% (15/30)	43.3% (13/30)	33.3% (10/30)	43.3% (13/30)	46.7% (14/30)
		p = 0.518^a^	p = 0.621^a^	p = 0.095^a^	p = 0.012^a^	p = 0.095^a^	p = 0.506^a^
Grade 1 Decreased 0.1% to <10% from baseline	Lithium	34.8% (8/23)	13% (3/23)	8.7% (2/23)	26.1% (6/23)	17.4% (4/23)	8.7% (2/23)
	Placebo	23.3% (7/30)	16.7% (5/30)	20% (6/30)	23.3% (7/30)	30% (9/30)	20% (6/30)
		p = 0.495^a^	p = 0.264^b^	p = 0.229^b^	p = 0.817^a^	p = 0.233^b^	p = 0.229^b^
Grade 2 Decreased 10 to <30% from baseline	Lithium	26.0% (6/23)	26.1% (9/23)	43.5% (10/23)	21.7% (5/23)	34.8% (8/23)	23% (6/23)
	Placebo	26.7% (8/30)	13.3% (5/30)	30% (9/30)	33.3% (10/30)	13.3% (4/30)	30% (9/30)
		p = 0.971^a^	p = 0.164^a^	p = 0.765^a^	p = 0.484^a^	p = 0.145^a^	p = 0.814^b^
Grade 3 Decreased ≥30 to <50% from baseline	Lithium	8.7% (2/23)	8.7% (2/23)	8.7% (2/23)	8.7% (2/23)	8.7% (2/23)	8.7% (2/23)
	Placebo	0%	0%	3.3% (1/30)	0%	13.3% (4/30)	3.3% (1/30)
		p = 0.202^b^	p = 0.202^b^	p = 0.418^b^	p = 0.202^b^	p = 0.493^b^	p = 0.418^b^
Grade 4 Decreased ≥50% from baseline	Lithium	0%	0%	0%	0%	0%	0%
	Placebo	0%	0%	0%	3.3% (1/30)	0%	0%
					p = 574^b^		
Creatinine not measured^c^	Lithium	0%	0%	0%	4.3% (1/23)	0%	0%
	Placebo	0%	10% (3/30)	3.3% (1/30)	3.3% (1/30)	0%	0%
			p = 0.197^b^	p = 0.574^b^	p = 0.687^b^		

*eGFR* estimated glomerular filtration rate, *MDRD* modification of diet in renal disease formula

^a^Chi-squared test

^b^One-sided Fisher’s exact test

^c^Participants did not attend the specific study visit

^d^Grading according to the Division of AIDS (DAIDS) Table for Grading the Severity of Adult and Pediatric Adverse Events (Version 2.0 November 2014)

## Discussion

We reported the renal safety of lithium co-administered with TDF as part of a 24 week randomised placebo-controlled trial. To the best of our knowledge we described the first safety data of co-administered lithium with TDF. We found that lithium and TDF co-administration did not increase the risk of renal impairment in HIV-infected patients with neurocognitive impairment and preserved renal function over a 24-week period.

NDI is a well-recognised early side effect of lithium administration. Lithium causes dysregulation of the aquaporin-2 water channels in the collecting ducts with impaired pro-urine concentration ability [13, 15]. Three patients in the lithium arm developed NDI which resolved with a lithium dose reduction. Lithium-induced nephrotoxicity has been long recognised, but the extent and risk factors required to frame a risk-benefit profile for patients has been much debated [16]. A recent population-based study in psychiatric patients with lithium exposure found that monthly eGFR decline was similar in the lithium and reference group after adjusting for co-morbidities, concomitant medication and episodes of lithium toxicity [17]. Our findings in a young cohort with no lithium toxicity episodes and limited treatment duration echo these findings.

Our study has several limitations. First, we reported on the safety of lithium dosed with TDF in a randomised placebo-controlled trial that was not powered for this endpoint. Second, we followed patients for 24 weeks and we can only make inferences about the short-term safety of concomitant lithium and TDF administration. Third, we may have missed more subtle markers of tubulopathy as we did not measure urine markers of tubulopathy. Fourth, only approximately half of participants had therapeutic lithium trough concentrations. We collected lithium trough concentrations as soon as participants arrived at the study site and despite best efforts, sample collection time for some participants was beyond 12 h. Last, we excluded patients with renal impairment and concomitant medication which may potentiate lithium toxicity.

We could not rule out nephrotoxicity of long-term concomitant treatment of TDF and lithium and future research should focus on the long-term follow-up of TDF-treated HIV-infected patients with lithium-treated bipolar disorder.

## Conclusions

We found that 24-week treatment of HIV-infected patients with lithium and TDF, preserved renal function and no episodes of lithium toxicity did not result in increased nephrotoxicity. To the best of our knowledge we described the first safety data of co-administered lithium with TDF. Our finding supports the renal safety of TDF-based ART in HIV-infected patients with bipolar disorder requiring lithium therapy as a mood stabiliser.

## Abbreviations

ART: antiretroviral therapy
DNA: deoxyribonucleic acid
eGFR: estimated glomerular filtration rate
GSK-3-β: glycogen synthase kinase-3-beta
HIV: human immunodeficiency virus
MDRD: modification of diet in renal disease
MRP-4: multi-drug resistance protein 4
NDI: nephrogenic diabetes insipidus
OATP-1: organic anion transporter-1
TDF: tenofovir disoproxil fumarate
